# Spatial distribution of Qinghai spruce forests and the thresholds of influencing factors in a small catchment, Qilian Mountains, northwest China

**DOI:** 10.1038/s41598-017-05701-6

**Published:** 2017-07-17

**Authors:** Wenjuan Yang, Yanhui Wang, Shunli Wang, Ashley A. Webb, Pengtao Yu, Xiande Liu, Xuelong Zhang

**Affiliations:** 1 Research Institute of Forest Ecology, Environment and Protection, Chinese Academy of Forestry, Beijing, 100091 China; 2 Academy of Water Resource Conservation Forests of Qilian Mountains in Gansu Province, Zhangye, 734000 China; 3 NSW Department of Primary Industries, Tamworth Agricultural Institute, Calala, NSW 2340 Australia

## Abstract

Forest restoration in dryland mountainous areas is extremely difficult due to dry climate, complex topography and accelerating climate change. Thus, exact identification of suitable sites is required. This study at a small watershed of Qilian Mountains, Northwest China, aimed to determine the important factors and their thresholds limiting the spatial distribution of forests of Qinghai spruce (*Picea crassifolia*), a locally dominant tree species. The watershed was divided into 342 spatial units. Their location, terrain and vegetation characteristics were recorded. Statistical analysis showed that the potential distribution area of Qinghai spruce forests is within an ellipse with the axes of elevation (from 2673.6 to 3202.2 m a.s.l.) and slope aspect (from −162.1° to 75.1° deviated from North). Within this ellipse, the forested sites have a soil thickness ≥40 cm, and slope positions of lower-slope, lower- or middle-slope, anywhere if the elevation is <2800, 2800–2900, >2900 m a.s.l, respectively. The corresponding mean annual air temperature at upper elevation boundary is −2.69 °C, while the mean annual precipitation at lower elevation boundary is 374 (331) mm within the small watershed (study area). The high prediction accuracy using these 4 factors can help to identify suitable sites and increase the success of afforestation.

## Introduction

Restoring the reduced forests of high mountains to protect the environment of dryland regions is of worldwide importance. Firstly, this is because the dryland regions cover one-third of the total land area of the world^1^. The dryland area ratio in China is even as high as 47.5%^2^, but concentrated in the northwest. Secondly, the high mountains are often important areas supplying vital water resources for dryland regions, where water scarcity is the major issue to be addressed^3^. Thirdly, the high mountains are also important habitat for most natural forests which supply numerous ecosystem services, including erosion control, timber production, biodiversity protection, carbon sequestration, flood mitigation, and so on. Therefore, increasing attention and efforts have been given to the mountain forests in dryland regions.

The area of mountain forests in dryland regions has been significantly reduced in the past due to deforestation to meet the increasing demand for timber and grazing with rapid population growth. For example, in the Qilian Mountains of northwest China, the forest cover has been reduced from 22.4% in the 1950s to 12.4% in the 1990s^4^. Similarly, African forests declined at an estimated rate of 4.0 million ha per annum between 2000 and 2005^5^. The forest area reduction is viewed as one important cause and indicator of environmental degradation, thus the current forestry policy is often directed to forest restoration programs for improving the environment and meeting the rapidly increasing demands on diverse forest ecosystem services. For example, Senegal, Uganda, Nepal, Indonesia, Bolivia, and Nicaragua have protected forests in many regions by transitioning toward decentralized forest management that allows local actors increased rights and responsibilities^6, 7^, and the Grain for Green Program and the Natural Forest Protection Program are examples from China^8^.

However, forest restoration on many mountain sites in the arid zone is extremely difficult, since forest growth is limited by the variable climate and site quality factors. These include severe drought limitation at lower elevations with very low precipitation and at sunny sites with stronger solar radiation, as well as cold limitation on higher elevation sites due to the decreased temperature. Furthermore, the high mountains in dryland regions are experiencing a more intense climate change. The arid region in central Asia has continued warming in the 20th century^9^, and the annual air temperature in Qilian Mountains has shown an upward trend in recent times, at a rate of 0.29 °C per decade^10^. The arid and semi-arid regions of the world are becoming more and more dry^11^, e.g., the precipitation in semi-arid regions of West Africa has continued to decrease since the 1960s, and the precipitation in Sahel region has been reduced by 20–40%^12^. This climate change will continue to modify the moisture and temperature conditions of all sites, with a consequence of changed site suitability for forest growth and forest spatial distribution at the landscape scale^13^. The distribution of South African biomes has been drastically altered by climatic change in the 21st century^14^. Potential ranges of 130 North American tree species will decrease in size by 58% in the next century affected by currently predicted change in climate^15^. Climate-induced forest response has significantly modified the spatial patterns of high-elevation forests in southern Siberia from 1960 to 2002^16^. Therefore, we need firstly to identify the important topographic factors influencing forest distribution; secondly to quantify the thresholds of these topographic factors; and thirdly to derive the limits of climatic factors (mainly precipitation and temperature) to give more precise and reliable guidance for forest restoration under stronger climate change in the high mountains of dryland regions.

The study area of this paper is the Qilian Mountains, headwaters of the Heihe River, the second largest inland river in China, and where Qinghai spruce (*Picea crassifolia*) is the dominant tree species accounting for 79.6% of the total forest area^17^. Many studies have been conducted on its hydrological impacts^18–20^. However, there are few studies up to now on the spatial distribution patterns of Qinghai spruce forests in the Qilian Mountains. The first study was implemented by Zhao *et al*.^21^ who determined the potential distribution area of Qinghai spruce forests in the Qilian Mountains by combining field observation and remote sensing data and using a species-habitat model. This method can be used to extract the spatial extent of environmental variables relevant to the distribution area of Qinghai spruce to determine the boundary function, and then make reasonable estimates about the habitat of Qinghai spruce^22–24^. However, remote sensing data cannot be used to distinguish the Qinghai spruce from other tree species, such as Qilian juniper (*Sabina przewalskii*), as accurately as field observations. Furthermore, the current distribution of Qinghai spruce forests is likely to be strongly influenced by human activities, so it may not accurately reflect the suitable habitat. Therefore, a comprehensive survey is required.

In this study, we took the example of the small watershed of Pailugou, where long-term ecohydrological studies have been carried out, to describe the spatial distribution of Qinghai spruce forests based on field investigations in the whole watershed, and to determine the main influencing factors and their thresholds.

## Results

### Spatial distribution of vegetation in small watershed

**Figure 1:**
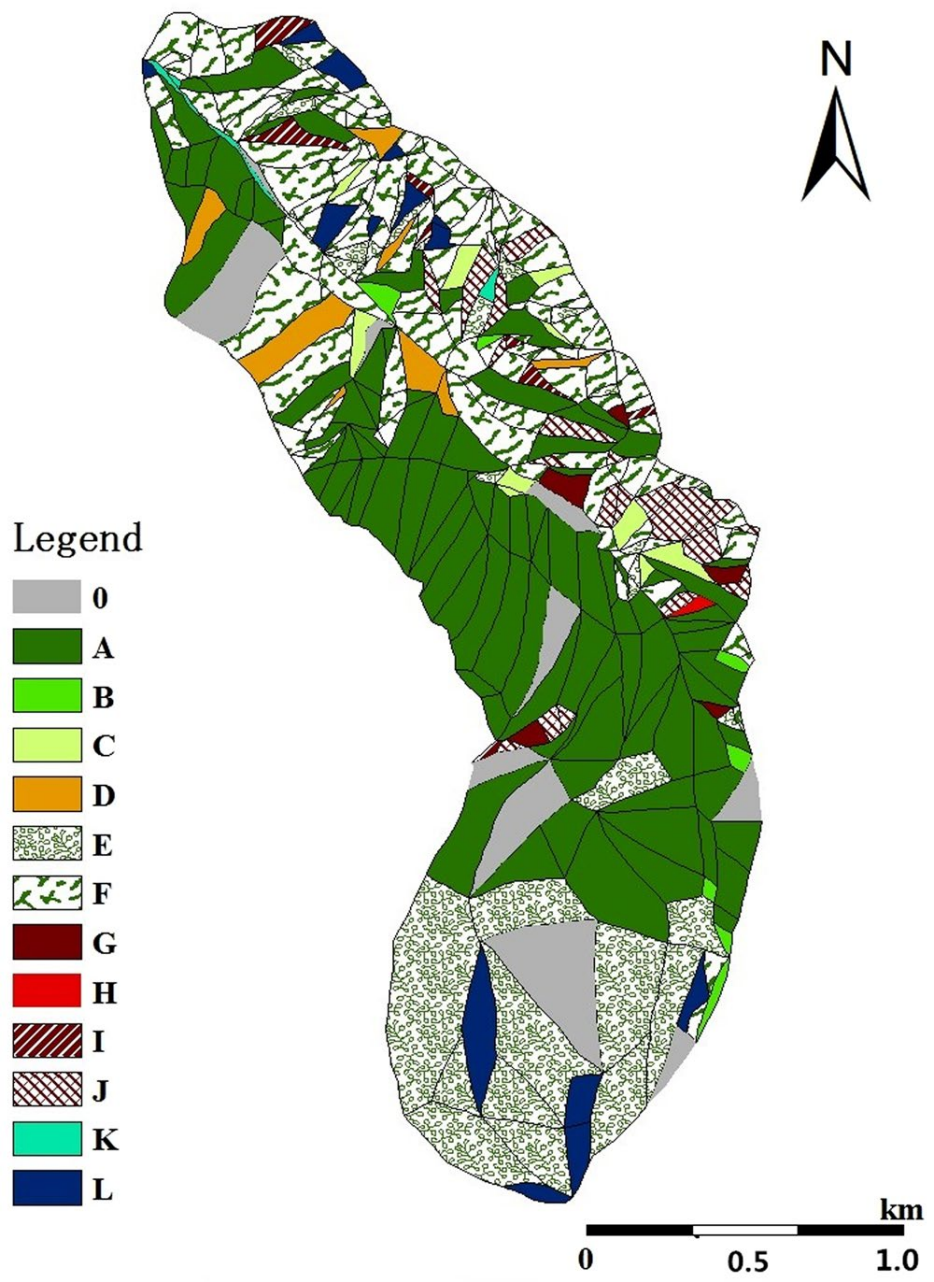
The distribution of vegetation cover types in Pailugou watershed (0 unmeasured units, (A)) Qinghai spruce forest, (B) Qinghai spruce woodland, (C) Qinghai spruce montane savanna shrub, (D) Qinghai spruce montane savanna grass, (E) shrub, (F) grassland, (G) *Sabina przewalskii* forest, (H) *S. przewalskii* woodland, (I) *S. przewalskii* montane savanna shrub, (J) *S. przewalskii* montane savanna grass, (K) watercourse, and (L) bare land. The legends are all the same in other figures thereafter). This map was generated using ArcGIS 10.3 software.

The small watershed of Pailugou was divided into 342 spatial units, but 11 units (accounting for 7.7% of the watershed area) were not investigated due to their difficult geographical location, such as extremely steep slopes or the impossibility of reaching the units. The remaining 331 units were therefore investigated and attributed to one of the eight main vegetation cover types (See Table 1 and the Study site and methods sections.). In addition, the forest, woodland, montane savanna shrub and montane savanna grass were subdivided according to the dominant tree species of Qinghai spruce or Qilian juniper (Table 1, Fig. 1).

**Table 1:** The vegetation cover types and their area ratio in the Pailugou watershed.

Vegetation cover type		Unit number	Area ratio to whole watershed/%	Vegetation cover type		Unit number	Area ratio to whole watershed/%
Forest	Qinghai spruce	100	31.9	Montane savanna shrub	Qinghai spruce	10	1.3
	Qilian juniper	5	1.2		Qilian juniper	8	0.6
Woodland	Qinghai spruce	7	0.8	Montane savanna grass	Qinghai spruce	8	1.6
	Qilian juniper	1	0.1		Qilian juniper	21	2.1
Shrub		31	19.2	Bare land		14	2.7
Grassland		123	30.6	Watercourse		3	0.2

It can be seen from Table 1 that there are 100 units forested with Qinghai spruce and 5 units forested with Qilian juniper; 8 woodland units including 7 of Qinghai spruce and 1 of Qilian juniper; 18 montane savanna shrub units including 10 Qinghai spruce montane savanna shrub and 8 Qilian juniper montane savanna shrub; 29 montane savanna grass units including 8 Qinghai spruce montane savanna grass and 21 Qilian juniper montane savanna grass; 31 shrub units and 123 grass units. In addition, there are 14 rocky bare land units and 3 watercourse units. There are no glaciers in the Pailugou watershed. The tree species of Qilian juniper is distributed only on sunny slopes, its quantity in the small watershed is less, with an area ratio (including forest, woodland and montane savanna) of just 4.0%, so we have not analyzed and evaluated its distribution in this paper.

**Figure 2:**
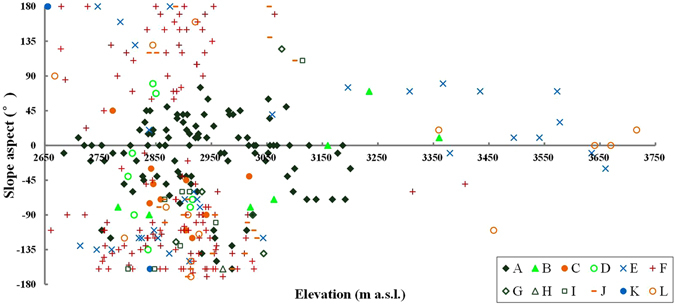
The vegetation distribution with relation to elevation and slope aspect in Pailugou watershed. This map was generated using Microsoft Excel 2010 software.

Using the mean elevation and slope aspect of spatial units as the X axis and Y axis respectively, the vegetation distribution pattern in the small watershed was plotted in Fig. 2. It can be seen that the Qinghai spruce forests are distributed within the elevation range of 2684–3201 m a.s.l., but mostly within 2800–3100 m a.s.l., accounting for 80% of its whole area. In the elevation range above 3200 m a.s.l., probably limited by the low temperatures, only two Qinghai spruce woodland units exist, with most of the spatial units being covered by shrubs.

We can also see from Fig. 2 that the forests are distributed within the slope aspect range from −160° to 75°, but firstly concentrated on the shady slopes (−45° to 45°), accounting for 73% of its whole area; then distributed on the semi-sunny slopes (−45° to −135°) and semi-shady slopes (45° to 135°), accounting for 19% and 5% of its whole area, respectively; and rarely (only 3%) distributed on the sunny slopes (135° to 180° and −135° to −180°).

The grassland is concentrated (94.5% of its whole area) within the elevation range of 2650–3000 m a.s.l., and by comparison is mostly distributed on sunny, semi-sunny and semi-shady slopes (accounting for 41.1%, 43.1% and 12.5% in area, respectively), but rarely on shady slopes (only 3.3%).

### The boundary of potential distribution area of Qinghai spruce forests

**Figure 3:**
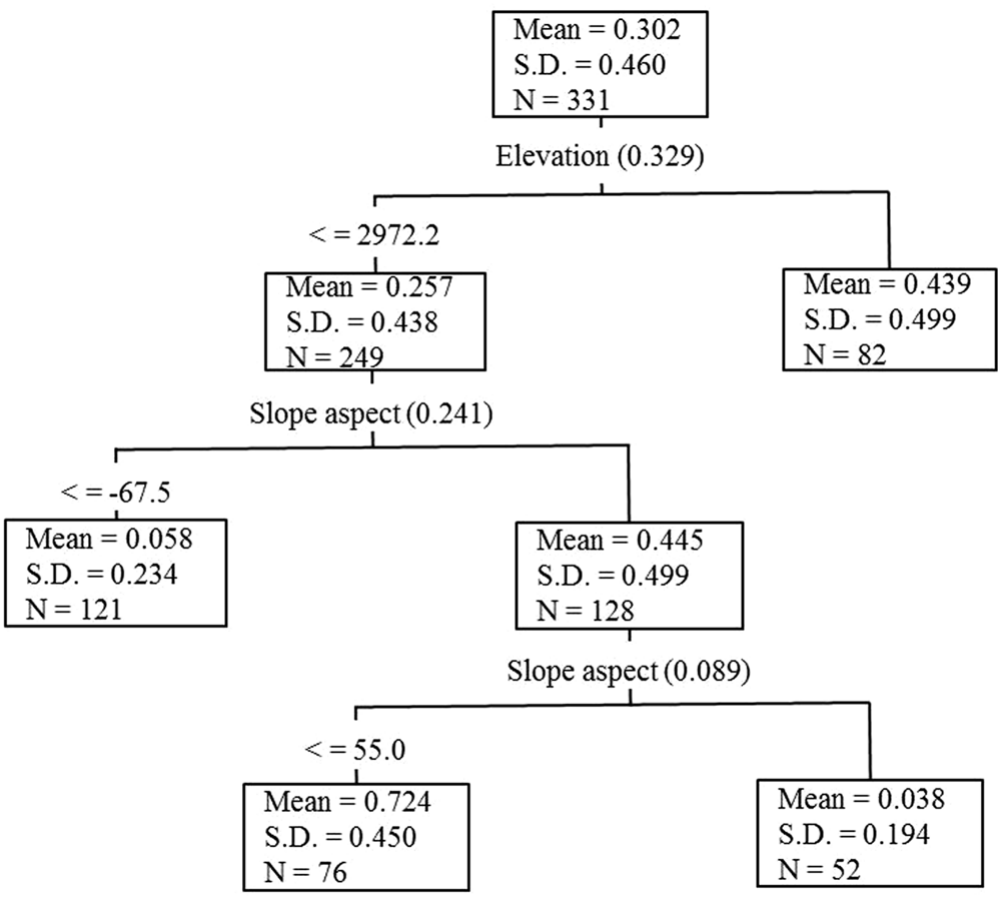
Regression tree predicting distribution of Qinghai spruce forests (and non-forest vegetation) from limiting factors (PRE = 0.659). Each node (square) is labeled with average forested ratio (mean), standard deviation (S.D.) and the number (n) of spatial units in that group. The model is read from top down until terminal nodes appear. Partial PRE values are presented in parentheses at each root node to split. This map was generated using SYSTAT 13 (Systat Software, San Jose, CA).

To identify the most important limiting factors, we fitted a regression tree for describing their relative influence on the prediction accuracy of the distribution of Qinghai spruce forests (and non-forest vegetation) in Pailugou watershed (Fig. 3). The optimum regression tree has three splits and four terminal nodes. The first split occurred at an elevation of 2972.2 m a.s.l., which suggested that the elevation is the most important factor in determining the distribution of Qinghai spruce forests. There were 82 spatial units with an elevation >2972.2 m a.s.l. and the forested ratio of these 82 units is 0.439 (here the forested unit is valued as 1, and the non-forested units is valued as 0). This single split can account for 32.9% of the total variation in forest distribution. Slope aspect was the second most important factor and it significantly impacts only those units with an elevation <2972.2 m a.s.l. and can explain 24.1% of the total variation in forest distribution. The units with both elevation <2972.2 m a.s.l. and a slope aspect >−67.5° were further split again on the basis of slope aspect, which explained 8.9% of the total variation in forest distribution. The fact that no additional splits were performed indicated that elevation and slope aspect are the two most important factors influencing the prediction accuracy of spatial distribution of Qinghai spruce forests. These two factors can jointly explain a large part of the variation in forest distribution, with 65.9% of proportional reduction in error (PRE).

**Figure 4:**
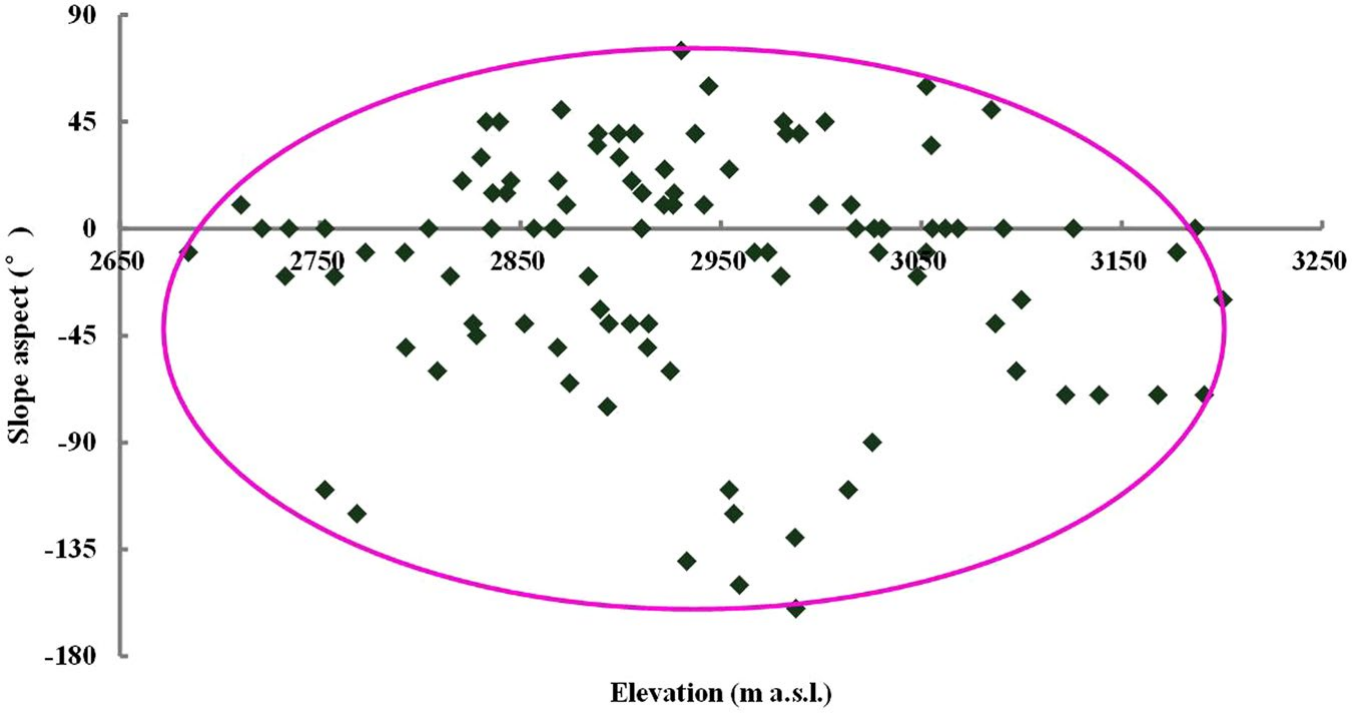
The ellipse shaped potential distribution area of Qinghai spruce forests with relation to elevation and slope aspect in Pailugou watershed. This map was generated using Microsoft Excel 2010 software.

Based on the result of regression tree that elevation and slope aspect are the most important factors, we connected the outlying points of Qinghai spruce forests in Fig. 2. Then it can be found that the potential distribution area is in the form of an ellipse (Fig. 4), within which nearly all Qinghai spruce units (including Qinghai spruce woodland, Qinghai spruce montane savanna shrub and Qinghai spruce montane savanna grass) are included, except just 2 Qinghai spruce woodland units. Therefore, we can assume this ellipse covers the potential distribution area of Qinghai spruce forests in the small watershed, if only the limiting factors of elevation and slope aspect are considered. The equation of the ellipse was fitted as below:

1 $$\frac{{(H-2937.92)}^{2}}{{264.31}^{2}}+\frac{{(A+43.53)}^{2}}{{118.58}^{2}}=1{R}^{2}=0.9967$$

where H is the elevation (m a.s.l.), A is the slope aspect (°).

According to the fitted ellipse equation, the center point of the ellipse was located at an elevation of 2937.9 m a.s.l. and slope aspect of −43.5°. This indicates that the potential distribution area of Qinghai spruce forests is not focused on the exact north-facing slope, but there is an obvious counterclockwise deflection of −43.5° from the north direction in its bilateral symmetry.

The lower and upper elevation limits of the potential distribution area of Qinghai spruce forests in the studied small watershed are 2673.6 m a.s.l. and 3202.2 m a.s.l., respectively. The slope aspect limits of the potential distribution area of forests are −162° in the counterclockwise direction and 75° in the clockwise direction from north at the elevation of 2937.9 m a.s.l., respectively. However, the limiting threshold of slope aspect varies with elevation. For example, it increases from −77° at an elevation of 2684 m a.s.l. to −162° in the counterclockwise direction from north at an elevation of 2937 m a.s.l., and correspondingly from −10° to 75° in the clockwise direction from north. However, the range of slope aspect of potential forest area does not increase further when the elevation further increases; in contrast, it decreases as shown in Fig. 4 and described by Equation (1).

### Factors limiting the Qinghai spruce forests within the potential distribution area

**Figure 5:**
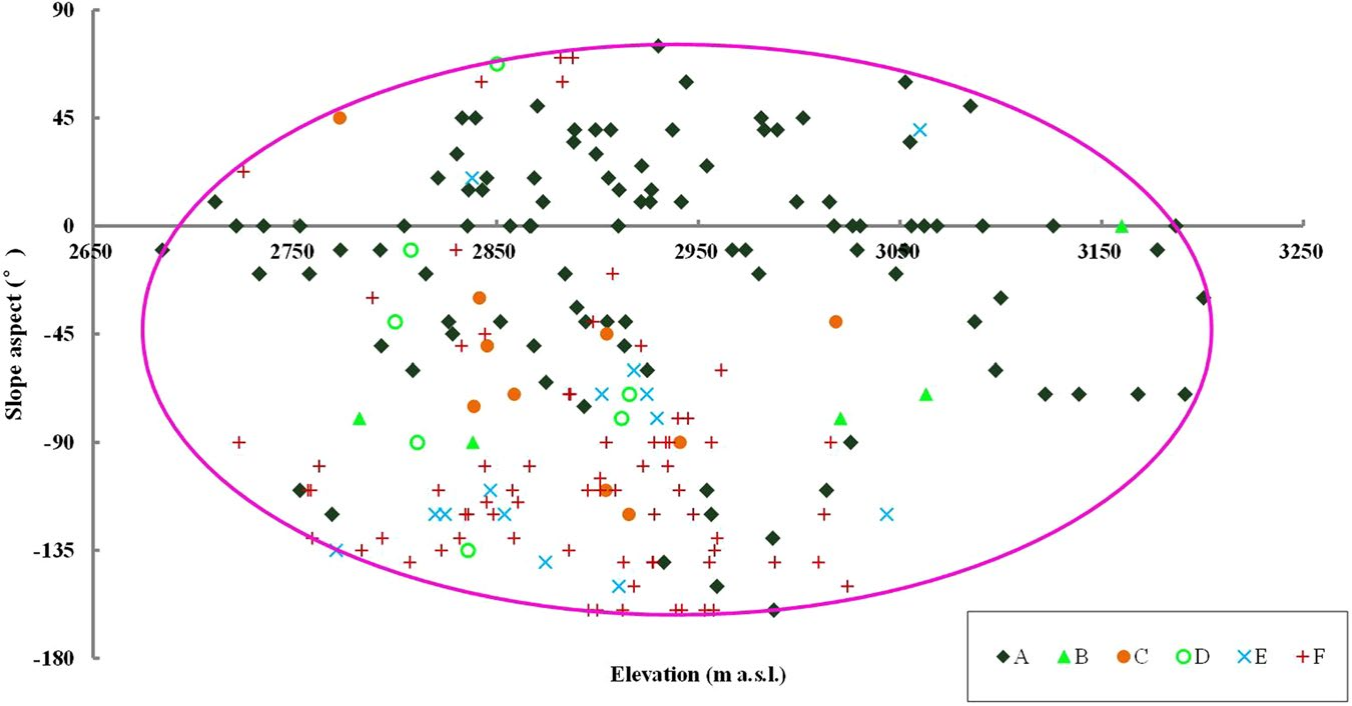
Composition of vegetated units of all vegetation types within the potential distribution area of Qinghai spruce forests. This map was generated using Microsoft Excel 2010 software.

**Figure 6:**
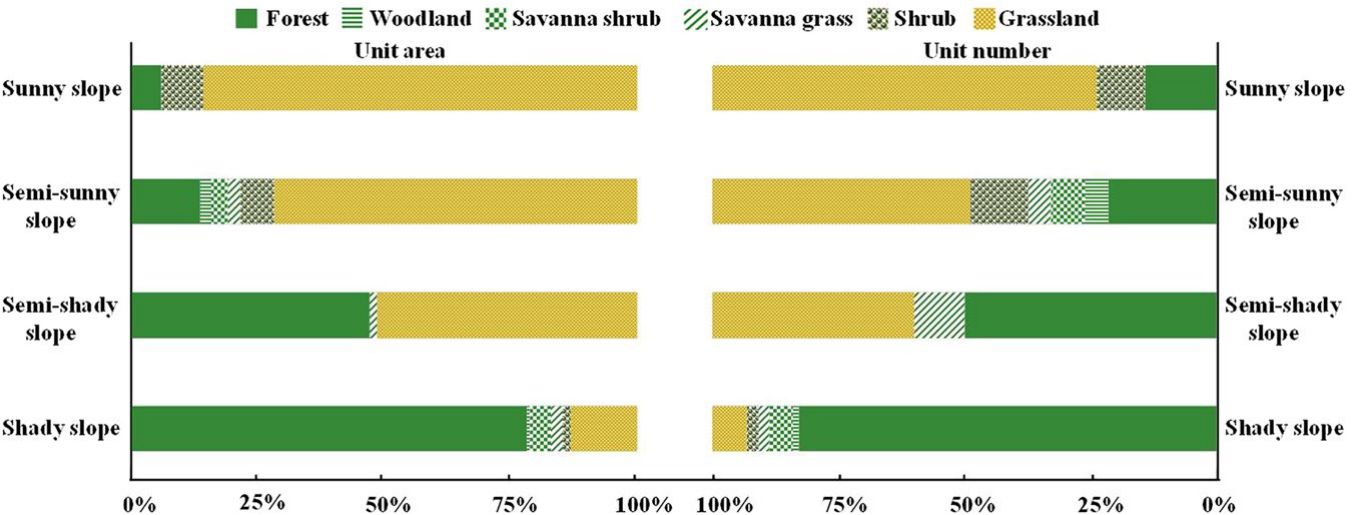
Ratios of spatial unit number and area of different vegetation types according to four slope aspects within the ellipse shown in Fig. 5. This map was generated using Microsoft Excel 2010 software.

Many non-forest vegetated units exist within the potential distribution area of Qinghai spruce forests (Fig. 5). These non-forest vegetation units are mostly shrub land or grassland on semi-sunny and sunny slopes within the elevation range of 2723–3060 m a.s.l.. The grassland and shrub land units are distributed within the slope aspect range from −60° to −160°, accounting for 84.9% and 88.2% of the total area of grassland and shrub land within the potential distribution area respectively, where there are few forested units accounting for only 16.7% of the total Qinghai spruce forests area. In contrast, the units on shady slopes are mainly covered by Qinghai spruce forests, with an area ratio of 78.1%, with other vegetation types accounting for just 21.9% of the total 88 units on shady slopes (Fig. 6). This indicates that other factors besides elevation and slope aspect, such as slope position, slope gradient and soil thickness, also limit the growth of Qinghai spruce forests within the ellipse, through their influence on the soil water condition.

**Figure 7:**
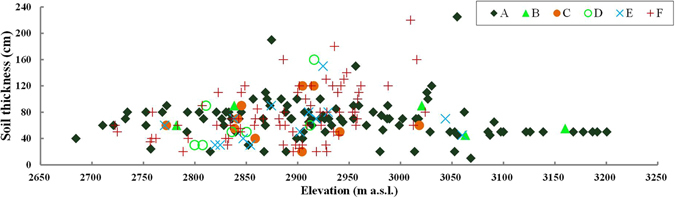
Soil thickness composition of all vegetated units within the potential distribution area of Qinghai spruce forests. This map was generated using Microsoft Excel 2010 software.

Soil thickness can affect the water-holding capacity of soil and then also the forest’s ability to resist drought stress; thus it should be an important factor limiting the forest distribution in dryland regions. From Fig. 7 it can be seen that the soil thickness of forested units varies within the range of 24–90 cm at elevations <2800 m a.s.l., within which just one forested unit has a soil thickness of 24 cm, while all others are ≥40 cm. In the elevation range of 2800–3060 m a.s.l., the soil thickness of forested units varies within the range of 20–225 cm, but mostly (90.9% in area) within the soil thickness ≥40 cm. At elevations >3060 m a.s.l., all units are forested, with a narrower soil thickness range of 10–65 cm. However, 94.8% of the area has soil of thickness ≥40 cm.

**Figure 8:**
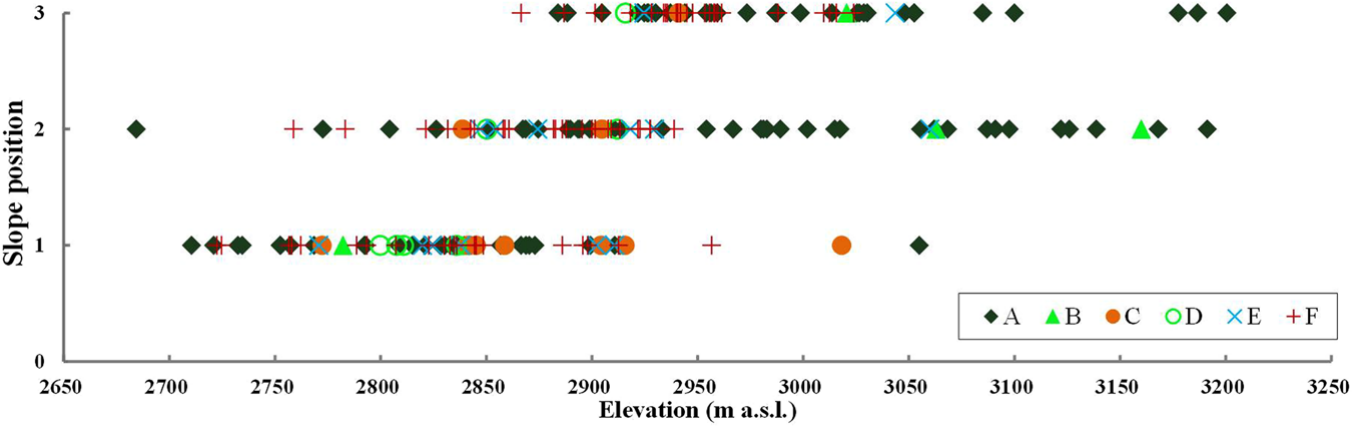
The distribution of all vegetated units within the potential distribution area of Qinghai spruce forests among the slope positions (1, 2 and 3 stands for lower slope, middle slope and upper slope, respectively). This map was generated using Microsoft Excel 2010 software.

To detect differences in forest distribution among slope positions, all the vegetated units were plotted in Fig. 8. At elevations <2800 m a.s.l., the Qinghai spruce forests are mainly distributed on the lower-slopes, with just 2 units on the middle-slope. Within the elevation range of 2800–2900 m a.s.l., the Qinghai spruce forests are also mainly distributed at the middle- and lower-slopes, accounting for 34.7% and 61.6% of the total area of Qinghai spruce forests within this elevation range, respectively. However, when the elevation is above 2900 m a.s.l., the Qinghai spruce forests are distributed throughout the hill slope.

**Figure 9:**
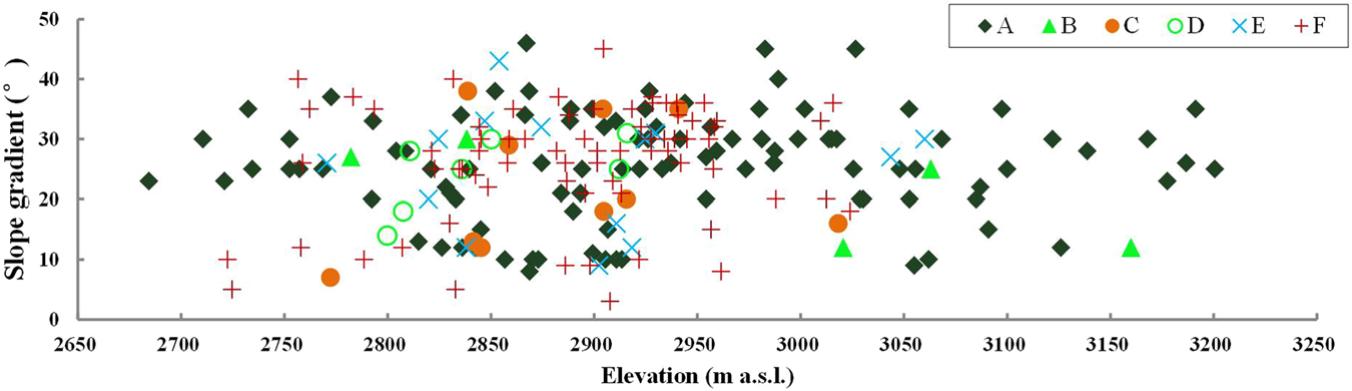
Slope gradient composition of all vegetated units within the potential distribution area of Qinghai spruce forests. This map was generated using Microsoft Excel 2010 software.

Slope gradient is also a possible factor influencing the forest distribution. At elevations <2800 m a.s.l., the forested units occupy most of the sites with a slope gradient of 20–37° (Fig. 9); while the gentler or steeper units are covered by grass or shrubs. This may be explained by the fact that the steeper slope gradient is favorable to generate more runoff and thus create drier site conditions to limit forest growth, while gentler slopes are favorable for the use of grassland for sheep grazing. At elevations >2800 m a.s.l., the forested units are scattered within the slope gradient range of 9–46°, almost the full variation range of slope gradient in the small watershed. This indicates that the slope gradient plays a somewhat limiting role for the forest distribution just at elevations <2800 m a.s.l., but not where elevations are >2800 m a.s.l..

To compare the influence of each factor on the spruce forests distribution, a principal components analysis was further conducted. The result showed that the first three principal components can account for 73.3% of the forest distribution. The first principal component (PC_1_), which was primarily comprised by elevation, could explain 30.9% of the forest distribution; while the second (PC_2_) and third principal component (PC_3_), which were primarily comprised by slope aspect and soil thickness respectively, could explain 22.2% and 20.2% of the forest distribution. This is consistent with the results of the above mentioned analysis.

## Discussion

### Forest distribution ranges of elevation and slope aspect

According to the results of this study, most spruce forests are distributed within the elevation range of 2684–3201 m a.s.l. and where slope aspects range from −160° to 75° in the small watershed. The potential distribution area of spruce forests follows the form of an ellipse. This ellipse has an elevation range of 2673.6–3202.2 m a.s.l., and changing borders of slope aspect with elevation, reaching the maximum range from −162.1° to 75.1° at the elevation of 2937.9 m a.s.l.. The most suitable elevation for Qinghai spruce is about 2950 m a.s.l., which agrees with the finding of Zhang^25^. Some other studies conducted in the Qilian Mountains have reported more or less different spatial distribution area of Qinghai spruce forests (Table 2). The reason for the higher bottom elevation border in our study is the limited elevation range (2642–3794 m a.s.l.) of the studied small watershed. We have observed that some forests do grow at some wetter sites on shady slopes or near valleys with lower elevations than the outlet of this small watershed (with elevation of 2642 m a.s.l.). Combined with the results of other studies, such as the study by Yang^26^ conducted in Xishui Nature Reserve, within which the small watershed is included, we can deduce that the bottom elevation border of Qinghai spruce forests in the study region of this paper is about 2430 m a.s.l.. The lowest bottom elevation border of 2351 m a.s.l. reported in the study of Xu^27^ was due to its more humid study location. As wind will be stronger near mountain summits, there is always an elevation belt without forests below the mountain summit. Therefore, the upper elevation border of forest distribution area is influenced by the highest elevation of mountains when it is not obviously above the upper tree line. In summary, the elevation range of 2430–3202.2 m a.s.l. for the Qinghai spruce forests in the study region of this paper agrees well with other studies.

**Table 2:** The spatial distribution of Qinghai spruce according to published studies.

Study region	Location	Study area/km^2^	Elevation range of study sites/m a.s.l.	Elevation range of forests (m a.s.l.)	Slope aspect range of forests	Literatures
Whole region of Qilian Mountains				2500~3300	Shady and semi-shady slope	Liu Xingcong.^29^
Dongxia forest region in Qinghai Province	101°35′–101° 54′E, 36°56′–37°15′N	161.86	2450~4348	2600~3100	Shady and semi-shady slope	Wang *et al*.^28^
Xishui Nature Reserve	100°03′–100°23′E, 38°32′–38°48′ N	732.49	2000~4000	2500~3200	−67.5°~112.5°	Yang *et al*.^26^
Portion of the Qilian Mountains	98°34′–101°11′E, 37°41′–39°05′N	10,009	2000~5500	2600~3400	−60°~96°	Zhao *et al*.^21^
Portion of the Qilian Mountains	93°23′–104°3′E, 36°2′–40°32′N		2200~5400	2351~3300	−110°~150°	Xu *et al*.^27^
Pailugou watershed	100°17′E, 38°32′N	2.74	2642~3794	2673.6 (2430)~3202.2	−162.1°~75.1° (−110°~75°)	In this study

However, the reported slope aspect ranges of Qinghai spruce forests obviously differ from each other. Compared with other studies, the slope aspect range is wider in the counterclockwise direction from north, but narrower in the clockwise direction from north in our study. These differences may be caused by the differences in geographic location and climate conditions, the size of study area and the elevation range of individual studies. In addition, the definition of forests used in our study is different from the landscape studies^26, 28^ which defined forest with a canopy density of ≥0.4, and from the studies^21, 27, 29^ dealing with the upper/lower tree line of Qinghai spruce. It seems that a study about the spatial distribution of Qinghai spruce forests with the same forest definition (not individual tree) and the same accurate data of forest area within the entire Qilian Mountains is required. In such a study, more attention should be given to the direct influences of climatic factors (water and temperature) and mankind activities (e.g., grazing) rather than only the geographic factors (elevation, slope aspect, etc.) on the forest distribution.

### Influence of climatic factors on the elevation range of forest distribution

The changing climatic conditions with elevation and slope aspect should be the main direct determinants for the spatial distribution of forests. With rising elevation, precipitation usually increases while air temperature decreases which leads to lower evapotranspiration and thus a higher amount of water available for plants^30–32^. This is vitally important for the spatial distribution of forest on the mountains in arid regions. The upper tree line is mainly determined by the low temperatures, while the lower tree line is mainly determined by the amount of precipitation^33^. This phenomenon was also observed for the broad-leaved species in the typical dry valley slopes of the Bhutan Himalaya^34^. The mean annual precipitation is 368 mm at the elevation of 2650 m a.s.l. and increases by about 4.95% per 100 m^18^; while the mean annual air temperatures is 0.5 °C at the base of the catchment (2650 m a.s.l.) with a decreasing rate of about 0.58 °C/100 m^35^ of elevation in the small watershed. In this study, the elevation range of Qinghai spruce forests in the small watershed (study region) varies from 2673.6 (2430) m a.s.l. to 3202.2 m a.s.l., which corresponds with mean annual air temperatures from 0.36 (1.37) °C to −2.69 °C, and annual precipitation from 372.4 (330.95) mm to 480.2 mm. The minimum annual precipitation for the Qinghai spruce forests determined in our study is similar to the results of Liu^29^ (300~500 mm), Zhao^36^ (300~620 mm) and Peng^22^ (300~442 mm). Therefore it can be concluded that the limiting threshold of mean annual precipitation at the bottom elevation border should be 372.4 mm in the studied small watershed, but 330.95 mm in the study region. The limiting threshold of mean annual air temperature at the upper elevation border of Qinghai spruce forests distribution area is −2.69 °C in the small watershed of Pailugou.

The elliptical relationship between slope aspect border and rising elevation in this study can only partly be explained by our current knowledge. The slope aspect can affect the amount of incident radiation and therefore further affect the temperature, evapotranspiration and thus also the water budget and the soil water available for plants. Compared with sunny slopes, the shading from mountain slopes decreases the incident radiation and increases the available soil water and moisture condition on shady slopes^37, 38^. This is a significant factor determining the spatial distribution of forests. For instance, Douglas fir in eastern Washington always occupies the shady northern slope of hills and is entirely absent on the sunny southern slope^39^. As a result of increasing annual precipitation with rising elevation, the slope aspect range of potential forest distribution area increases with rising elevation. For example, in the counterclockwise direction from north, it increases from −77° at the elevation of 2684 m a.s.l. to −162° at the elevation of 2937 m a.s.l., where the maximum slope aspect range is reached and the annual mean air temperature and precipitation are −1.16 °C and 422.8 mm, respectively. However, the decrease of slope aspect range of potential forest distribution area with further rising elevation after the maximum slope aspect range cannot be explained by the limitation of annual precipitation. Annual precipitation is not a limiting factor for forest growth at elevations above 2900 m a.s.l. where the annual precipitation amounts to 415.3 mm. This also cannot be explained by the limitation of annual air temperature, since the temperature will increase when the slope aspect is close to south. The potential reasons can be the thin soil on sunny and semi-sunny slopes at higher elevation, or the higher competition of Qilian juniper as a sunny tree species against the Qinghai spruce^40^, or the mankind induced land use that the warm sunny slopes are preferred to be used as grassland for cattle or sheep. Further studies are needed to validate these hypotheses.

### Influence of geographic factors on the distribution of Qinghai spruce forests

Soil thickness influences the existence and growth of forests, because it strongly affects the plant-available water amount, root development, tree recruitment^41^, and the buffering capacity against drought etc.^42^. Meerveld *et al*.^43^ found that the spatial differences in soil thickness appear responsible for the observed spatial differences in species distribution. In our study, most Qinghai spruce forests (92.6% in area) grow in soil of thickness ≥40 cm. Yang’s^44^ study also found that soil thickness above 40 cm is better for the growth of *Picea korainesis*. This indicates that soil thickness above 40 cm can store enough available water for Qinghai spruce forests to survive the dry periods. Yet there is one north-facing forested unit with a soil thickness of 10 cm in the elevation range above 3060 m a.s.l., which means that the precipitation there and other possible water input from upper-slope is enough to meet the water demand of Qinghai spruce as a tree species with a shallow root system^45^. Among the 29 units with soil thickness above 100 cm, 19 units are grassland located on slope aspects ranging from −60° to −160°, and only 3 units are covered by Qinghai spruce forests. This tells us that the limitation of soil thickness for forest distribution is much weaker than the limitation of slope aspect. This is supported by the study of Du^46^ which showed that *Picea balfouriana* on shady and semi-shady slopes with soil thicknesses of 45 and 38 cm grow better than trees on the sunny and semi-sunny slopes with soil thicknesses of 62 and 55 cm.

The effect of slope position on the distribution of forests is mainly caused by the downwards lateral movement of surface runoff and interflow in soil layers, which makes the upper-slope drier than the lower-slope^47, 48^. In this study, the Qinghai spruce forests are mainly (97.4% in area) distributed on lower-slopes at elevations below 2800 m a.s.l., and concentrated (96.3% in area) at the middle- and lower-slope positions within the elevation range of 2800–2900 m a.s.l., due to the greater availability of soil water. The distribution of Qinghai spruce forests on various slope positions is more variable with increasing elevation. This can be explained by the improved soil moisture at upper- and middle-slopes due to the increased precipitation and decreased temperature and evaporation with rising elevation. This indicates that the slope position does not play a limiting role for forest distribution when the elevation is above 2900 m a.s.l., where the precipitation is adequate. This finding is similar to some previous studies^49, 50^. There were very few units with a slope position of lower-slope when the elevation is above 3000 m a.s.l., which is due to the geographic condition of the studied small watershed, and possible limitations of the survey methods used.

Slope gradient can affect the existence of forests by altering many environment factors, such as the solar radiation, soil texture, soil erosion, soil moisture, and soil nutrition. Generally, gentle slopes provide better soil moisture and nutrients^51^. In this study, the range of slope gradients upon which Qinghai spruce forests grow is as wide as 9–46°, but 85% of Qinghai spruce forests grow on moderate and steep slopes with a gradient of ≥15°. This may be a result of land use decisions as gentle slopes are preferred to be used as grassland for grazing. However, it seems that the slope gradient is not a limiting factor for the distribution of Qinghai spruce forests, which is similar to the result of Niu’s study^52^.

The statistical analysis showed that elevation and slope aspect are the main factors influencing the spatial distribution of Qinghai spruce forests, while the soil thickness and slope position can also play a role. Therefore, using Equations (2) and (3), the prediction accuracy of the distribution of spruce forests (and non-forest vegetation) by considering the limiting factors and their thresholds determined in this study was calculated. The first limiting factor is elevation within the range of 2673.6–3202.2 m a.s.l.; the second limiting factor is slope aspect which changes with elevation as the ellipse (Equation (1)) described; the third limiting factor is the soil thickness of ≥40 cm; the fourth limiting factor is the slope position (lower-slope in elevation range <2800 m a.s.l., both middle- and lower-slope within the elevation range of 2800–2900 m a.s.l., and the whole slope in the elevation range >2900 m a.s.l.). We can see from Table 3 that the accumulated prediction accuracy in area is 76.2% (It would be 88.3% if the 7.7% uninvestigated area and the 4.0% of Qilian juniper forests are excluded.) when considering all the four limiting factors.

**Table 3:** The accumulated prediction accuracy of the distribution of Qinghai spruce forests (and non-forest vegetation) by considering the limiting factors.

	Actual spruce forest percentage	Elevation	Slope aspect	Soil thickness	Slope position
Unit number	29.2%	37.7%	65.5%	71.4%	74.6%
Unit area	31.9%	55.8%	71.5%	73.9%	76.2%

As pointed out by Guisan and Zimmermann^53^, it is uncontroversial that in many regions of the world a species may be absent from a site, at a local or regional level, not only due to geographical barriers or climatic events, but also because of the simple and sometimes dramatic effects of human activities aimed at transforming forests into crop or pasture lands. So we assume that disturbance by human activities is also a factor influencing the distribution of Qinghai spruce forests in the Pailugou watershed. Such activities include past deforestation and forest fires, and increasing and maintaining grassland for grazing. Liu^23^ found that some suitable sites for forests were converted to other land uses, such as pastures or settlement. An extreme example is the Loess Plateau where Qinghai spruce had the maximum extent covering the whole Loess Plateau between 8000–6000 years B.C., but the increasing intensity of human activities since about 2000 years B.C. led to the disappearance of spruce forests^54^. However, no detailed studies have been conducted to quantify the effects of human disturbance on the spatial distribution of spruce forests, and this remains as a hotspot for future studies.

The spatial distribution of forest is determined by the integration of many factors. In this study, the main influencing factors were analyzed and the thresholds of some of these factors were determined. The potential distribution area of Qinghai spruce forests and its quantitative expression using the factors of elevation and slope aspect was determined. Because of the limited number of spatial units, the small area and limited elevation range of the watershed studied, relationships to express the multiple factor interactions have not been established. In addition, this study has attempted to interpret the spatial distribution of Qinghai spruce forests using the annual precipitation and temperature based on the meteorological data of the small watershed, and to determine the thresholds of climatic factors, but soil moisture has not been included, though it is likely to have a more direct impact than precipitation. In addition, no tree physiological studies (in terms of water and temperature) have yet been used to explain the spatial distribution of Qinghai spruce forests. Integrated studies of ecology and physiology, as well as further field investigations and controlled experiments, are needed to understand and quantify the impacts of individual factors and their interactions on the spatial distribution of Qinghai spruce forests under a changing climate and the influence of human activities.

In this study, we selected a representative arid area in the Qilian Mountains, northwestern China, and the typical site factors (elevation, slope aspect, soil thickness, slope position and slope gradient) were considered. The factors influencing the spatial distribution of Qinghai spruce forests, which was the dominant tree species in Qilian Mountains, were identified. These research methods and qualitative conclusions may provide a reference and guidance for studies of the distribution of coniferous forests in other dryland mountains. However, due to the small area of this watershed studied, the quantitative conclusions and especially the threshold values of influencing factors in this study are not necessarily applicable to different coniferous forests in other dryland mountains.

## Conclusions

This study in a small watershed of Qilian Mountains in northwest China showed that the spatial distribution of Qinghai spruce forests in dryland mountainous areas is controlled not only by large-scale climatic factors (mainly annual precipitation and air temperature), but also by the mid-scale topographic factors of elevation and slope aspect, and by the small-scale factors of slope position and soil thickness. In the small watershed with limited area, the control of climatic factors is reflected as an elevation range for forest distribution, i.e., a minimum annual precipitation determining the lower elevation boundary and a minimum annual air temperature determining the upper elevation boundary. In the small watershed studied, the potential distribution area of spruce forests is determined by slope aspect and elevation, in the form of an ellipse with these two factors as the axes of ellipse. Further control is exerted by soil thickness and slope position within the potential distribution area of spruce forests, i.e., the existence of forest on some sites is determined by the soil-thickness-related soil water storage capacity and by the slope-position-related lateral slope runoff input. The prediction of suitable sites for forestation in dryland mountainous areas under climate change can obviously be improved by including the non-climatic factors. The procedure of forestation site selection considering the important factors and their thresholds determined in this study can be used as a reference for other dryland mountainous areas.

## Study site and Methods

### Study region

The Qilian Mountains is located in the northeast of Qinghai province and the western border of Gansu province (94°10′–103°04′ E, 35°50′–39°19′ N). The mountain range is 800 km long from east to west and 200–400 km wide from north to south. Most peaks are 4000 to 5000 m a.s.l., with the highest, Shule Mountain, having an elevation of 5808 m a.s.l.. The Qilian Mountains form the headwaters of several inland rivers, including the Heihe River, which is the second largest inland river in northwest China. The water resources supplied by the Heihe River are so important that it is called ‘the lifeblood’ for the 5 million people living in the Hexi Corridor^55^. The Heihe river flows through the provinces of Qinghai, Gansu and Inner Mongolia, converges at the East- and West-Juyan Lake, with a length of 821 km, a drainage area of 130,000 km^2^, and an annual runoff of 24.5 × 10^8^ m^3^ ^56^. There has been a serious water use conflict in the Heihe basin between the users in the upper- and middle- and down-stream reaches, and between different sectors such as agriculture, industry, forestry and domestic consumption. This has led to a drastic runoff reduction downstream and the drying up of more than 30 tributaries as well as the terminal lakes^21^. Maintaining a stable water supply from the head-water areas of the Qilian Mountains and balancing the competing water uses are key factors in the sustainable development of the region.

### Study site

**Figure 10:**
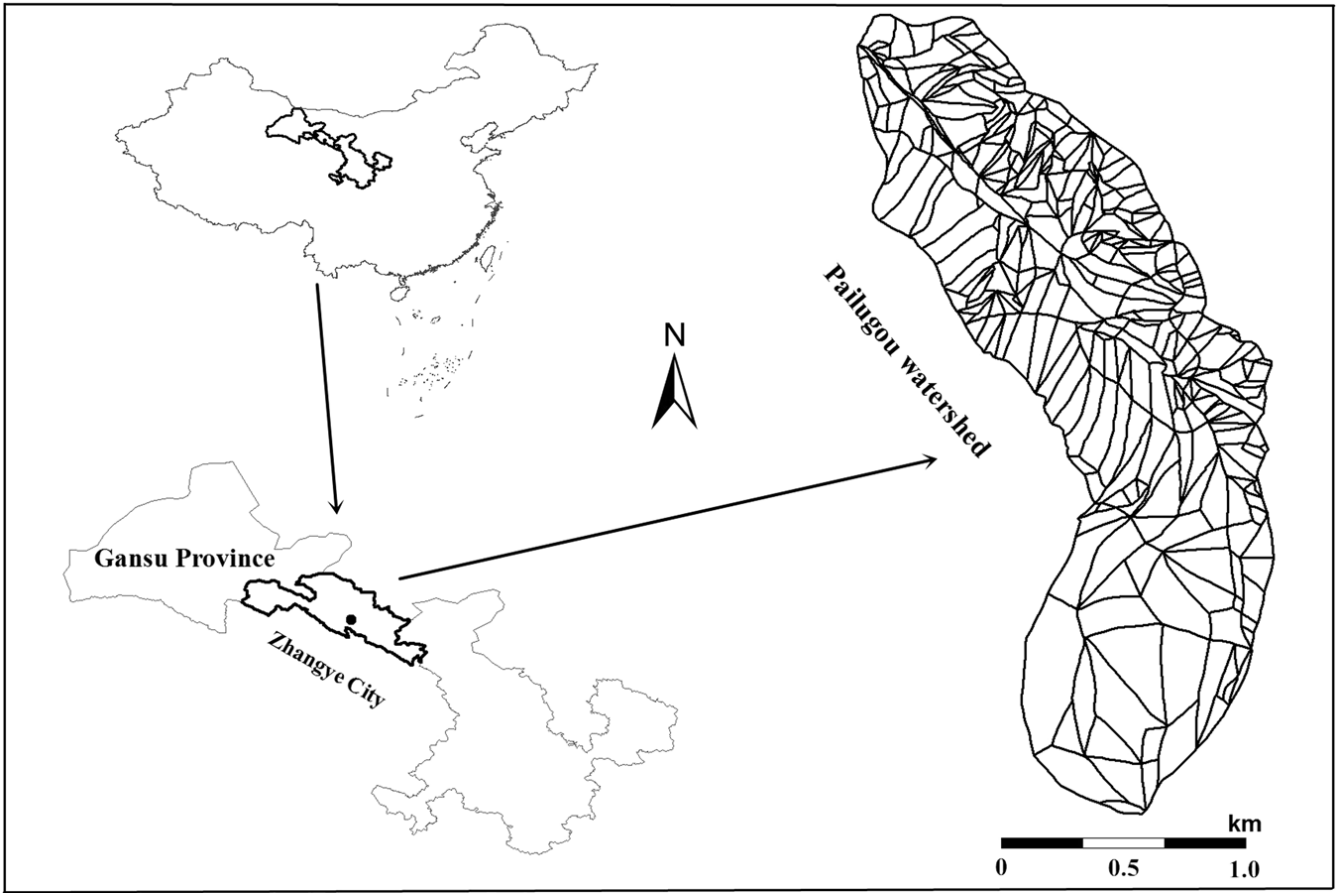
Location of the small watershed of Pailugou in the Qilian Mountains of northwest China, and the division of spatial units in the small watershed. This map was generated using ArcGIS 10.3 software.

The selected small watershed of Pailugou is located at the central north edge of the Qilian Mountains (100°17′E, 38°24′N), and close to Zhangye City, Gansu province (Fig. 10). Its total area is 2.74 km^2^, with an elevation range of 2642–3794 m a.s.l.. Based on the weather station located at the elevation of 2650 m a.s.l. and close to the outlet of this small watershed from 1994 to 2003, the mean annual precipitation is 368 mm while the mean annual air temperature is 0.5 °C^57^. About 60% of the annual precipitation occurs in the summer period from July to September^18^. Permanently and seasonally frozen soils are widespread at the middle and higher elevation ranges in the small watershed. The main parent material is calcareous rock. The main soil type is gray coarse soil, with a coarse texture, an intermediate organic matter content, and a pH range of 7–8.

### Study methods

#### Field Measurement

To describe the spatial distribution of forest/vegetation in the small watershed of Pailugou, it was divided into 342 spatial units in 2003, with the criteria of having the same or similar topography, vegetation type, soil properties and climate. The location of each unit was measured with a global positioning system (GPS), and its terrain characteristics (e.g., slope gradient, slope aspect, elevation) were recorded by a compass and an elevation meter. The soil thickness of each spatial unit was determined by soil profile method. The tree canopy density, the coverage of shrub layer, grass layer, and moss layer were measured in each spatial unit by line transect method. The tree height and diameter at breast-height (DBH) were measured for every individual Qinghai spruce with a DBH ≥ 5.0 cm.

#### Slope aspect valuation

In order to quantitatively analyze the impact of slope aspect of the spatial units on the distribution of spruce forest, the north-facing slope was evaluated as 0°; whereas other slope aspects were evaluated as their angle deviating from north. For example, the aspects of east and southeast were evaluated clockwise as 90° and 135°; and the west and southwest were evaluated in the counterclockwise as −90° and −135°.

### Classification of vegetation and forest types

In this study, each spatial unit was attributed to one of the following eight main vegetation cover types: forest, woodland, montane savanna shrubs, montane savanna grass, shrubs, grassland, bare land and watercourse. Forest was defined as the land with a tree canopy density of ≥0.2, woodland was defined as the land with a tree canopy density of ≥0.1 but <0.2, montane savanna shrubs was defined as the land dominated by shrubs but with a tree canopy density of <0.1, montane savanna grass was defined as the land dominated by grasses but with a tree canopy density of <0.1, shrubs was defined as the land dominated by shrubs without trees, and grassland was defined as the land dominated by grasses without trees, referring to the National Forest Inventory (NFI) Technical Regulations (2014) published by the State Forestry Administration of China.

### Statistical methods

Statistical analyses including regression tree and principal components analysis were performed by STSTAT 13 (Systat Software, San Jose, CA) and SPSS Statistics 17.0. We chose the least squares method for regression tree and varimax method for principal components analysis. In this study, regression tree was used to identify the most important factors (such as elevation, slope aspect, slope position, slope gradient and soil thickness) influencing the Qinghai spruce forest distribution. Principal components analysis was used to estimation the relative influence of the factors for the spruce forest distribution.

### Accuracy of the forest/vegetation distribution prediction using limiting factors

To evaluate the prediction of Qinghai spruce forests (and non-forest vegetation) distribution by considering the effects of limiting factors (starting from elevation, then adding others in the order of slope aspect, soil thickness, slope position, and so on) and their thresholds (determined later in this paper), the accuracy in both unit number and unit area were calculated using the following equations:

2 $${A}_{un}=(NI+NO)/N$$

3 $${A}_{ua}=(AI+AO)/A$$

where, *A*_*un*_ (*A*_*ua*_) is the accuracy of unit number (unit area); *NI* is the number of units of Qinghai spruce forests which are correctly predicted as spruce forest by considering the limiting factors; *NO* is the number of units of non-spruce forest which are correctly predicted by considering the limiting factors; similarly, *AI* (and *AO*) is the area of units of Qinghai spruce forests (no-spruce forest) which are correctly predicted; *N* (*A*) is the total unit number (area) in the small watershed (equals to 342 and 2.74 km^2^ respectively).
